# ZIP8 exacerbates collagen-induced arthritis by increasing pathogenic T cell responses

**DOI:** 10.1038/s12276-021-00591-1

**Published:** 2021-04-01

**Authors:** Jung-Ah Kang, Ji-Sun Kwak, Sang-Heon Park, Kyu-Young Sim, Seul Ki Kim, Youngnim Shin, In Jung Jung, Jeong-In Yang, Jang-Soo Chun, Sung-Gyoo Park

**Affiliations:** 1 School of Life Sciences, Gwangju Institute of Science and Technology, Gwangju, 61005 Republic of Korea; 2 Cell Logistics Research Center, Gwangju Institute of Science and Technology, Gwangju, 61005 Republic of Korea; 3 Infectious Disease Research Center, Korea Research Institute of Bioscience & Biotechnology (KRIBB), Daejeon, 34141 Republic of Korea; 4 National Creative Research Initiatives Center for Osteoarthritis Pathogenesis, Gwangju Institute of Science and Technology, Gwangju, 61005 Republic of Korea; 5 College of Pharmacy, Seoul National University, Seoul, 08826 Republic of Korea

**Keywords:** Autoimmunity, Immunological disorders

## Abstract

Zinc is a trace element that is essential for immune responses. Therefore, changes in cellular zinc levels in specific immune cells may influence inflammatory autoimmune diseases, such as rheumatoid arthritis (RA). However, the regulation of zinc mobilization in immune cells and its role in the pathogenesis of RA are not fully understood. Thus, we investigated the roles of zinc transporters in RA pathogenesis. We demonstrated that ZIP8 was specifically upregulated in CD4^+^ T cells that infiltrated the inflamed joint and that ZIP8 deficiency in CD4^+^ T cells abrogated collagen-induced arthritis. ZIP8 deficiency dramatically affected zinc influx in effector T cells and profoundly reduced T cell receptor (TCR)-mediated signaling, including NF-κB and MAPK signaling, which are pathways that are involved in T helper (Th) 17 cell differentiation. Taken together, our findings suggest that ZIP8 depletion in CD4^+^ T cells attenuates TCR signaling due to insufficient cellular zinc, thereby reducing the function of effector CD4^+^ T cells, including Th17 cells. Our results also suggest that targeting ZIP8 may be a useful strategy to inhibit RA development and pathogenesis.

## Rheumatoid arthritis: zinc spurs autoimmune response

Increased uptake of zinc by T cells may fuel the poorly controlled immune response that wreaks havoc on joint tissues in rheumatoid arthritis (RA). Zinc is important for immune function, and zinc levels within cells are tightly modulated by numerous importer and exporter proteins. Researchers led by Sung-Gyoo Park of the Seoul National University and Jang-Soo Chun of the Gwangju Institute of Science and Technology in South Korea set out to examine how these proteins influence the immune dysfunction associated with RA. Working with a mouse model of RA, they determined that levels of the zinc-importing protein ZIP8 are increased within inflamed joint tissues and in a key subset of T cells infiltrating those tissues. This ZIP8-mediated zinc uptake in turn activates and stimulates proliferation of these T cells. Drugs that target ZIP8 could counter the joint damage seen in RA.

## Introduction

Rheumatoid arthritis (RA) is a chronic inflammatory autoimmune disease that primarily affects joint tissues. This disease is characterized by synovial inflammation, cartilage destruction, and bone erosion^1,2^. The molecular mechanisms of RA pathogenesis are not fully understood, but the onset and progression of RA are known to be associated with various genetic factors. For example, the human leukocyte antigen allele *HLA-DRB1* in the major histocompatibility locus was the first RA risk locus identified; this allele regulates disease progression by affecting the T cell receptor (TCR) repertoire and/or the presentation of antigens to self-reactive T cells^3,4^. Alleles of other genes, such as *CD28*, *TRAF1*, *PTPN22*, and *CTLA-4* are also known to increase the risk of RA development. These RA-associated alleles are related to immune cell functions, and many are associated with the regulation of T cell responses^3,4^.

During synovial inflammation in RA pathogenesis, various leukocytes infiltrate the synovial compartment via the increased expression of adhesion molecules and chemokines in the endothelium, leading to the production of large amounts of chemokines and proinflammatory cytokines in inflamed synovial tissues^2^. Among the infiltrating cells, CD4^+^ T cells are necessary for RA onset^5,6^, and CD4^+^ T cell depletion suppresses autoantibody production and reduces the disease severity of collagen- or antigen-induced arthritis in rodent models^6,7^. Moreover, therapeutic strategies to block T cell costimulation are very effective against both the early and advanced disease stages of RA^8,9^. CD4^+^ T cells and macrophages are the main cell types that infiltrate the RA synovium^5,6^. Subsequent to the discovery of interleukin (IL)-17-producing CD4^+^ T cells in the RA synovium, studies using animal models have revealed that T helper (Th)17 cells are key players in various autoimmune and inflammatory diseases^10,11^. In addition to the critical involvement of Th17 cells, RA has been considered to be a Th1-dependent disease. However, experimental deficiency of Th1-promoting cytokines, such as IL-12 and interferon (IFN)-γ, has been shown to increase Th17 cell differentiation and paradoxically exacerbate RA in animal models^12^.

Zinc is a trace element that is essential for life, and organisms have evolved processes to strictly maintain zinc homeostasis^13^. In mammalian cells, zinc homeostasis is primarily regulated by membrane zinc transporters in the Slc30a (ZNT) exporter family and the Slc39a (ZIP) importer family^14^. The ZNT family, which consists of ten members in mammals (ZNT1–ZNT10), mediates the efflux of zinc from cells or the influx of zinc from the cytosol to intracellular vesicles. In contrast, the 14 members of the ZIP family in mammals (ZIP1–ZIP14) promote the influx of zinc from the extracellular space or intracellular vesicles into the cytoplasm. Previous reports have indicated that serum zinc levels are significantly lower in RA patients than in healthy controls^15,16^. In addition, zinc has been suggested to be an anti-inflammatory element^17^. In the immune system, zinc deficiency induces thymic atrophy and lymphopenia and reduces immune responses by regulating the functions of various immune cells, including macrophages, neutrophils, natural killer cells, B cells, and T cells^18^. Previous reports have indicated that ZIP8-mediated zinc import negatively regulates macrophage activation^19^, whereas ZIP10-mediated zinc import positively affects macrophage-mediated immune responses^20^. In addition, it has been reported that ZIP9 and ZIP10 are crucial for B cell receptor signaling cascades and affect B cell activation^21,22^. In T cells, ZIP6 affects TCR signaling cascades^23^, and ZIP8 is involved in cytokine production by activated T cells^24^. In summary, these reports delineate the specific roles of individual zinc transporters in regulating immune cell functions. Additionally, proinflammatory cytokines have been shown to alter the expression levels of zinc transporters, thereby decreasing serum zinc levels^25^.

Here, we investigated the possible role of specific zinc transporter-mediated zinc mobilization in RA pathogenesis. In the present study, we analyzed the expression levels and functions of ZIP and ZNT family members in T and B cells and macrophages. We found that the zinc transporter ZIP8 was specifically upregulated in inflamed synovial cells. Among the immune cells that infiltrated the inflamed joint tissues, we observed that synovial but not peripheral CD4^+^ T cells highly expressed ZIP8 on the cell surface. We further characterized the role of ZIP8 in CD4^+^ T cell functions and found that ZIP8 was crucial for regulating the functions of infiltrating CD4^+^ T cells during the onset and progression of collagen-induced arthritis (CIA).

## Materials and methods

### Mice and CIA

*Slc39a8*^+/−^, *Mtf1*^+/−^, and *Mt1*^−/−^*Mt2*^−/−^ mice were created as described previously^26,27^. *Slc39a8*^f/f^ mice were crossed with *Lysm-Cre* or *CD4-Cre* Tg mice (The Jackson Laboratory) to generate macrophage- or CD4 T cell-specific conditional knockout (cKO) mice (*Slc39a8*^f/f^;*Lysm-Cre* and *Slc39a8*^f/f^;*CD4*-*Cre* mice, respectively) on a C57BL/6 background. Each mouse line was backcrossed with DBA/1 J mice to generate the corresponding mice on a DBA/1 J background. All mice were maintained under pathogen-free conditions and used in accordance with protocols approved by the Animal Care and Ethics Committees of the Gwangju Institute of Science and Technology. CIA was induced according to a standard protocol^28^. Cumulative CIA incidence (score 1) was evaluated on the indicated days after the first immunization. All other CIA parameters were evaluated after the mice were sacrificed on day 36 or 39 after the first immunization. CIA severity was evaluated using a clinical score (grade 0−4) of paw swelling. Joint tissues were fixed, decalcified with 0.5 M EDTA, embedded in paraffin, and sectioned at a thickness of 5 μm. Synovitis was evaluated by Safranin-O and hematoxylin staining, and synovial inflammation (grade 0−4) was scored as previously described^28^. The pannus was visualized by H&E staining and quantified by scoring (grade 0−4)^28,29^. Cartilage destruction was visualized by Safranin-O staining and quantified by scoring cartilage extracellular matrix loss (score 0: normal; 1: slight reduction; 2: moderate reduction; 3: severe reduction; and 4: absent).

### Immunohistochemistry and immunofluorescence microscopy

Mouse joint tissues were sectioned at a thickness of 5 μm for immunohistochemical staining. Antigens were retrieved by incubating the joint sections at 60 °C overnight with sodium citrate buffer (10 mM sodium citrate, 0.05% Tween 20, pH 6.0). The sections were blocked with 1% bovine serum albumin in phosphate-buffered saline (PBS) and then incubated with primary antibodies, including rabbit anti-ZIP8 (Santa Cruz), rabbit anti-metal-regulatory transcription factor-1 (MTF1), anti-metallothionein 1 (MT1)/MT2 (Novus Biologicals), rabbit anti-matrix metalloproteinase 3 (MMP3), anti-receptor activator of NF-κB ligand (RANKL), and anti-Ki67 (Abcam). The Dako REAL Envision detection system was used for chromogenic color development. Tartrate-resistant acid phosphatase (TRAP)-positive osteoclasts were examined using a TRAP kit (Sigma-Aldrich) and counted in regions containing pannus–cartilage and pannus–bone interfaces^29^. ZIP8-expressing cells in the synovial tissues of CIA mice or primary cultures of total synovial cells isolated from CIA mice were identified by double immunofluorescence labeling with vimentin for fibroblast-like synoviocytes (FLSs), CD11b for macrophages, CD4 for T cells, and B220 for B cells. The following primary antibodies were used: mouse anti-CD4 (Abcam), rat anti-B220 (eBioscience), rat anti-CD11b (Abcam), rabbit anti-ZIP8 (Santa Cruz), and mouse anti-vimentin (BD Pharmingen). Blood vessels in synovial tissues were detected with rat anti-CD31 antibodies (Dianova). ZIP8 in isolated CD4^+^ T cells was analyzed by immunofluorescence microscopy using rabbit anti-ZIP8 antibodies (Alomone Labs).

### Isolation and analysis of total synovial cells

Total synovial cells were isolated from the knee joints of nonimmunized (NI) or CIA mice^29^. Synovial tissues were minced and digested in collagenase (Worthington, 2 mg/mL) for 4 h at 37 °C. Total cells were subjected to mRNA extraction for qRT-PCR analysis, primary culture to identify ZIP8-expressing cells (described above), and flow cytometric analysis to identify ZIP8-expressing infiltrated cells (described below).

### Zinc assays

Synovial fluid was collected from both knees of NI and CIA mice by joint aspiration^30^. The collected fluid was measured by the ‘pipette-dialing’ technique^30^. The volume collected was not significantly different between NI and CIA mice. The collected synovial fluid was mixed with 100 μl of PBS, and samples were cleared by centrifugation at 15,000*g* for 20 min. The levels of zinc were determined by using a zinc quantification kit (Abcam) according to the manufacturer’s instructions. To assess zinc influx in isolated primary naive (CD62L^high^CD44^low^) CD4^+^ T (Tnaive) cells and effector memory (CD62L^low^CD44^high^) CD4^+^ T (Tem) cells, the cells were incubated with 1 μg/mL Fluo-4 AM (cell permeant, Thermo) in PBS at 37 °C. After being washed with PBS, the cells were resuspended in PBS containing 45 μM ZnCl_2_. The intensity of the cellular dye was measured using a fluorescent plate reader (Molecular Devices) every 5 s for a total of 65 s. Intracellular zinc was measured using the probe ZnAF-1F (Santa Cruz). The sections of cartilage tissues were treated with 10 μM probe for 60 min at 37 °C. The sections were then washed with PBS, and zinc distribution images were acquired with a fluorescence microscope^26^.

### Enzyme-linked immunosorbent assay (ELISA) analysis of autoantibody production

Type II collagen-specific antibodies were measured by ELISA (Immunology Consultants Lab) according to the manufacturer’s instructions^29^.

### Flow cytometry

Thymocytes, splenocytes, and lymphocytes were isolated from *Slc39a8*^*f/f*^ and *Slc39a8*^*f/f*^;*CD4-Cre* mice as previously described^29^. The following antibodies were purchased from eBioscience for cell staining: Alexa Fluor 488-conjugated anti-mouse CD4 (GK1.5), fluorescein isothiocyanate (FITC)-conjugated anti-mouse TCRβ (H57-597), FITC-conjugated anti-mouse CD8 (53-6.7), peridinin chlorophyll protein cyanine 5.5 (PerCP Cy5.5)-conjugated anti-mouse CD25 (PC61.5), allophycocyanin (APC)-conjugated anti-mouse CD4 (GK1.5), APC-conjugated anti-mouse B220 (RA3-6B2), APC-conjugated anti-mouse IL-17A (eBio17B7), PerCP Cy5.5-conjugated anti-mouse CD62L (MEL-14), PerCP Cy5.5-conjugated anti-mouse IFN-γ (XMG1.2), and phycoerythrin-conjugated anti-human/mouse CD44 (IM7). Live cells were stained for 1 h at 4 °C and then fixed in 2.5% (wt/vol.) paraformaldehyde. To analyze activated CD4^+^ T cells from *Slc39a8*^*f/f*^ and *Slc39a8*^*f/f*^;*CD4-Cre* mice, the cells were activated for the indicated times with 5 μg/mL of anti-mouse CD3 (17A2) and anti-mouse CD28 (145-2C11) antibodies (both from Bio X Cell), after which the cells were stained with the indicated antibodies. For intracellular cytokine staining, isolated splenic cells or lymph node cells were stimulated for 6 h with phorbol myristate acetate (50 ng/mL) and ionomycin (500 ng/mL) in the presence of GolgiPlug (BD Pharmingen)^29^. Data were collected using a FACSCanto II (BD Biosciences) and analyzed with FlowJo software (BD Biosciences).

### In vitro differentiation of Th17 cells

CD4^+^ T cells were isolated from the spleens of *Slc39a8*^*f/f*^ and *Slc39a8*^*f/f*^;*CD4-Cre* mice using an EasySep^TM^ Mouse CD4^+^ T cell Isolation Kit (Stem Cell). For in vitro differentiation of CD4^+^ T cells into Th1 cells, isolated cells (2 × 10^5^) were cultured with anti-mouse CD3 antibodies (5 μg/mL), anti-mouse CD28 antibodies (5 μg/mL), IL-12 (20 ng/mL), IL-2 (20 ng/mL), and anti-IL-4 antibodies (10 μg/mL). For in vitro differentiation of CD4^+^ T cells into Th17 cells, isolated cells (2.5 × 10^5^) were cultured with anti-mouse CD3 (5 μg/mL) and anti-mouse CD28 (5 μg/mL) antibodies, IL-6 (100 ng/mL), TGF-β (5 ng/mL), and anti-IFN-γ (10 μg/mL) and anti-IL-4 (10 μg/mL) antibodies. IFN-γ- or IL-17A-producing cells were evaluated by flow cytometry^29^.

### Cytokine analysis

CD4^+^ T cells were isolated from *Slc39a8*^f/f^ and *Slc39a8*^f/f^;*CD4*-*Cre* mice on day 39 after the first immunization. To determine protein levels, the BD Cytometric Bead Array, mouse Th1/Th2/Th17 Cytokine Kit (BD Biosciences), and a FACSCanto II flow cytometer (BD Biosciences) were used to measure the secreted cytokines IL-2, IL-6, IL-10, IL-17A, IFN-γ, and tumor necrosis factor (TNF)-α. The mRNA levels of the indicated cytokines were determined by quantitative RT-PCR (described below).

### Proliferation assay

Tnaive and Tem cells (5 × 10^4^) isolated from *Slc39a8*^f/f^ and *Slc39a8*^f/f^;*CD4*-*Cre* mice were stimulated with anti-mouse CD3 (5 μg/mL) and anti-mouse CD28 (5 μg/mL) antibodies for 72 h. At 60 h after activation, [^3^H] thymidine was added to measure Tnaive and Tem cell proliferation. After 12 h, the incorporation of [^3^H] thymidine into the nuclear DNA of activated Tnaive and Tem cells was measured. Tnaive and Tem cells were also stained with 5 μM CellTrace Violet (CTV) (Thermo) for 5 min, and labeling was stopped by the addition of RPMI 1640 medium (HyClone) supplemented with 10% fetal bovine serum (HyClone). After several washes, the CTV-labeled cells were incubated for 48–72 h. Data were collected using a FACSCanto II (BD Biosciences) and analyzed with FlowJo software.

### Quantitative RT-PCR and western blot analysis

The PCR primers used and experimental conditions were described previously for ZIP and ZNT family members^26^ and various cytokines^29^. Transcript levels were determined by quantitative qRT-PCR. The following antibodies were used for western blotting: rabbit anti-ZIP6 (Thermo), rabbit anti-ZIP8 (Santa Cruz), mouse anti-GAPDH (Santa Cruz), mouse anti-lymphocyte-specific protein tyrosine kinase (LCK) (Santa Cruz), mouse anti-CD4 (Santa Cruz), mouse anti-phospho-TCRζ (Thermo), rabbit anti-src homology 2 domain-containing protein tyrosine phosphatase 1 (Shp-1) (Cell Signaling), rabbit anti-phospho-extracellular signal-regulated kinase (ERK) (Cell Signaling), anti-phospho-c-Jun N-terminal kinase (JNK) (Cell Signaling), mouse anti-inhibitor of κBα (IκBα) (Santa Cruz), mouse anti-phospho-IκBα (Cell Signaling), rabbit anti-protein kinase B (AKT) (Cell Signaling), and rabbit anti-phospho-AKT (Cell Signaling).

### Microarray analysis

Gene expression data were obtained from the study by Woetzel et al.^31^, as deposited in the Gene Expression Omnibus DataSets database at the National Center for Biotechnology Information (NCBI) (http://www.ncbi.nlm.nih.gov/gds/). The data can be found at accession numbers [GDS5401] (‘Berlin’ data), GDS5402 (‘Leipzig’ data), and GDS5403 (‘Jena’ data).

### Statistical analysis

For statistical comparisons of experimental groups, the datasets were examined by the Shapiro-Wilk test for normality and Levene’s test for homogeneity of variance. Nonparametric data based on the ordinal grading of mouse experimental groups (i.e., clinical score, synovitis, pannus formation, and OARSI grade) were compared using the Mann–Whitney *U* test. Parametric data collected from two independent experimental groups were evaluated by a two-tailed *t*-test. For comparisons of three or more groups, one-way analysis of variance (ANOVA) with a post hoc Bonferroni test was used. For the incidence of CIA, the chi-square (*χ*^2^) test was used. Each *N* number indicates the number of biologically independent samples or the number of mice per group. Significance was accepted at a probability level of 0.05 (*P* < 0.05). The bars represent the SEM or SD for parametric data and the calculated 95% confidence interval (95% CI) for nonparametric data.

## Results

### ZIP8 is specifically upregulated in the inflamed joint tissues of CIA mice and human RA patients

**Fig. 1:**
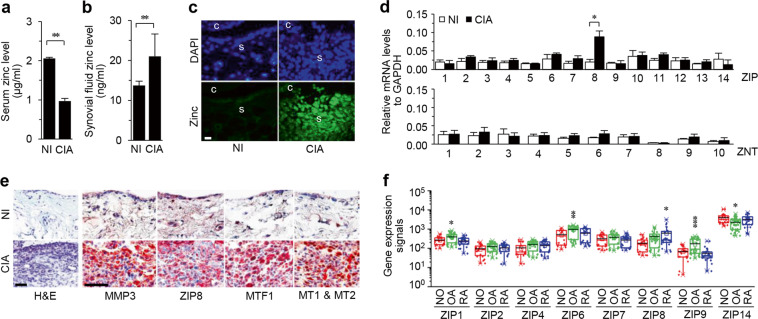
ZIP8 is upregulated in the inflamed joint tissues of CIA mice and RA patients. **a**, **b** Zinc levels in the serum (**a**) and knee joint synovial fluid (**b**) of CIA mice and nonimmunized (NI) control mice (*N* = 10 mice per group). **c** Representative images of cellular zinc levels in the joint tissues of CIA and NI mice (*N* = 5 mice per group). c, cartilage; s, synovium. **d** Relative mRNA levels of ZIP family (zinc importer) and ZNT family (zinc exporter) members in total synovial cells were quantified by qRT-PCR analysis (*N* = 6 mice per group). The error bars represent ±SD, and unpaired two-tailed Student’s *t*-tests were performed to determine differences between groups (**a**–**d**). **e** Representative images of immunostaining for MMP3, ZIP8, MTF1, and MT1/MT2 in the synovium of CIA and NI mice (*N* = 5 mice per group). **f** Microarray analysis of the indicated ZIP family members in synovial tissues from patients with rheumatoid arthritis (RA; *N* = 33), patients with osteoarthritis (OA; *N* = 26), and healthy individuals (HE; *N* = 20). Microarray data were obtained from the Gene Expression Omnibus database at the National Center for Biotechnology Information (NCBI). The box indicates the 25th and 75th percentiles, with the centerline representing the mean; significance was analyzed by one-way ANOVA followed by Bonferroni’s post hoc comparison. ^*^P < 0.05; ^**^P < 0.005; ^***^P < 0.0005; ns, not significant. Scale bars: 50 μm.

The serum zinc levels of RA patients are significantly lower than those of healthy individuals^15,16^. We also found significantly lower serum zinc levels in CIA mice than in NI control mice (Fig. 1a). However, CIA mice exhibited significantly increased zinc levels in synovial fluid (Fig. 1b) and synovial tissues (Fig. 1c) compared with those of control mice. This result indicates an alternation in zinc homeostasis under CIA conditions. To investigate the possible mechanisms of zinc mobilization into the inflamed joint tissues of CIA mice, we analyzed the mRNA levels of all ZIP and ZNT family members in total synovial cells. We observed specific upregulation of ZIP8 (Fig. 1d). Consistent with this finding, the ZIP8 protein level was markedly increased in the CIA synovium, in which the RA synovial marker MMP3 was detected (Fig. 1e). We additionally found upregulation of the zinc-dependent transcription factor MTF1 and the MTF1 target protein MTs^32^ in the CIA synovium (Fig. 1e). Analysis of gene expression microarray datasets^31^ from the Gene Expression Omnibus database (Fig. 1f) revealed that ZIP8 mRNA was also significantly upregulated in the synovial tissues of RA patients.

### ZIP8 regulates CIA in mice

**Fig. 2:**
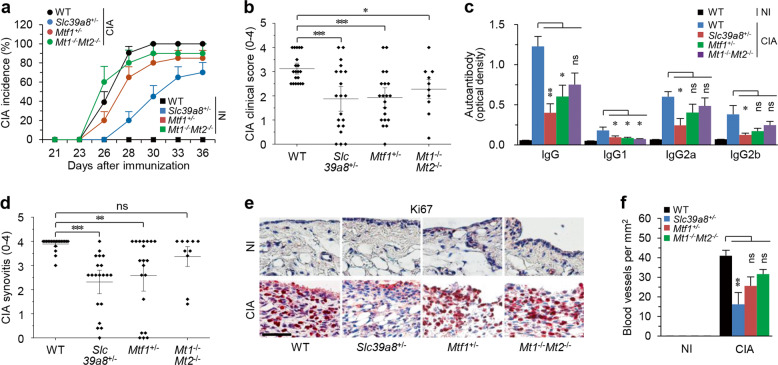
ZIP8 regulates CIA in mice. **a** Cumulative CIA incidence (score 1) on the indicated days after the first immunization of WT, *Slc39a8*^*+/−*^, *Mtf1*^*+/−*^, and *Mt1*^−/−^*Mt2*^−/−^ mice. Mean ± SEM are shown. **b** Severity of CIA in the indicated CIA mice, as assessed by measuring the clinical score. **c** Type II collagen-specific autoantibody production under NI and CIA conditions in the sera of the indicated mice. **d** Scoring of synovial inflammation (synovitis) in the indicated CIA mice. **e** Representative images of Ki67 staining to detect proliferating cells (*N* = 8 mice per group). Scale bars: 50 μm. **f** Number of blood vessels in the synovium of the indicated mice under NI and CIA conditions. The numbers of mice used were as follows: 20 WT, *Slc39a8*^*+/−*^, and *Mtf1*^*+/−*^ mice and 10 *Mt1*^−/−^*Mt2*^−/−^ mice (**a**, **b**, **d**). Eight mice per group were used in the experiments presented in (**c**, **e**, and **f**). All parameters in (**b**–**f**) were determined on day 36 after the first immunization. The values are presented as mean ± 95% CI and were assessed with the Mann-Whitney *U* test (**b**, **d**); the values are presented as mean ± SEM. and were assessed with ANOVA and Bonferroni’s post hoc comparison (**d**, **f**). ^*^P < 0.05, ^**^P < 0.005, ^***^P < 0.0005. ns: not significant.

Next, we investigated the potential role of the zinc–ZIP8–MTF1–MT axis in the pathogenesis of CIA using mice engineered with haplodeficiency of ZIP8 (*Slc39a8*^+/−^) or MTF1 (*Mtf1*^+/−^), or double deficiency of MT1 and MT2 (*Mt1*^−/−^*Mt2*^−/−^)^26^. We used heterozygous *Slc39a8*^+/−^ and *Mtf1*^+/−^ mice because homozygous deletion of *Slc39a8* or *Mtf1* is embryonic lethal^33,34^, and we used *Mt1* and *Mt2* double-knockout (KO) mice^26,32^ because MT1 and MT2 may have redundant properties, being almost identical in their structures and functions^32^. Compared with wild-type (WT) littermates, *Slc39a8*^+/−^ mice exhibited profound inhibition of CIA incidence, and *Mtf1*^+/−^ and *Mt1*^−/−^*Mt2*^−/−^ mice exhibited less inhibition than *Slc39a8*^+/−^ mice (Fig. 2a). Similarly, the reductions in CIA severity (clinical score, Fig. 2b), paw thickness (Supplementary Fig. 1a, b), and type II collagen-specific autoantibody production (Fig. 2c) were notable and significant in *Slc39a8*^+/−^ mice, whereas *Mtf1*^+/−^ and *Mt1*^−/−^*Mt2*^−/−^ mice exhibited reduced or nonsignificant inhibition of these CIA parameters.

Inflammatory arthritis (to which CIA is classified) is also characterized by synoviocyte proliferation with synovial hyperplasia, synovitis with immune cell infiltration, and angiogenesis in synovial tissues^1,2,5^. These hallmarks of CIA were also significantly inhibited in *Slc39a8*^+/−^ mice and to a lesser degree in *Mtf1*^+/−^ and *Mt1*^−/−^*Mt2*^−/−^ mice (Fig. 2d−f and Supplementary Fig. 1c, d). Pannus formation and its invasion into adjacent cartilage and bone are important steps in the erosion of cartilage and bone during RA pathogenesis. These processes are mediated by the actions of TRAP-positive osteoclasts, which are regulated by the expression of RANKL^1,2,5^. Consistent with this finding, under CIA conditions, *Slc39a8*^*+/−*^ mice exhibited significant reductions in pannus formation (Supplementary Fig. 2a), RANKL expression (Supplementary Fig. 2b), the differentiation of TRAP-positive osteoclasts in the pannus of the bone–cartilage interface (Supplementary Fig. 2c) and cartilage destruction (Supplementary Fig. 2d), whereas these CIA hallmarks were less apparent in *Mtf1*^+/−^ and *Mt1*^−/−^*Mt2*^−/−^ mice.

### ZIP8 is predominantly expressed in infiltrated CD4^+^ T cells

**Fig. 3:**
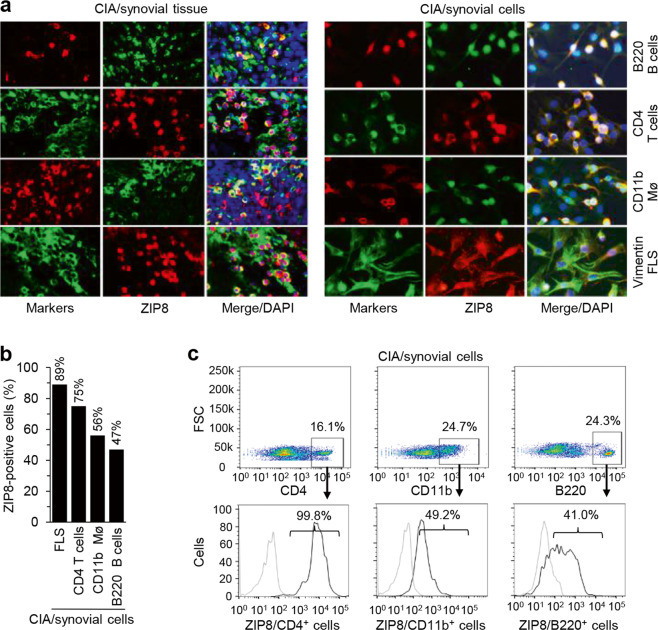
Among the infiltrated immune cells, ZIP8 is predominantly expressed in CD4^+^ T cells. **a**, **b** Typical immunofluorescence microscopic images of DAPI, ZIP8, and markers for B cells (B220), T cells (CD4), macrophages (CD11b), and fibroblast-like synoviocytes (FLSs, vimentin) in synovial tissues (left) and primary cultures of total synovial cells isolated from CIA mice (right) (**a**). The percentage of ZIP8-positive cells was determined by immunofluorescence microscopic analysis of primary cultures of total synovial cells isolated from CIA mice (**b** *N* = 5 mice). **c** Flow cytometric analysis of ZIP8 expression on CD4^+^ cells, CD11b^+^ cells, and B220^+^ cells from total synovial cells isolated from the knee joints of CIA mice. The data shown in (**a**, **c**) are representative of five independent experiments.

Immunofluorescence analysis of the synovial tissues of CIA mice or primary total synovial cells isolated from these mice revealed that ZIP8 was upregulated in multiple cell types in CIA mice, including vimentin-positive FLSs, B220-positive B cells, CD4-positive T cells, and CD11b-positive macrophages (Fig. 3a, b). We also quantified the number of ZIP8-positive cells among the infiltrated immune cells in the synovial tissues of CIA mice. Flow cytometric analysis revealed that almost all (99.8%) CD4^+^ T cells highly expressed ZIP8 on the cell surface, whereas less than half of CD11b^+^ macrophages and B220^+^ B cells were positive for ZIP8 expression (Fig. 3c). Moreover, ZIP8 was highly upregulated in activated CD4^+^ T cells but not in activated B220^+^ B cells or CD11b^+^ macrophages (Supplementary Fig. 3a–c). Based on the prevalent upregulation of ZIP8 in CD4^+^ T cells, we next focused on the role of zinc mobilization in CD4^+^ T cells during CIA pathogenesis.

### ZIP8 depletion in T cells inhibits CIA in mice

**Fig. 4:**
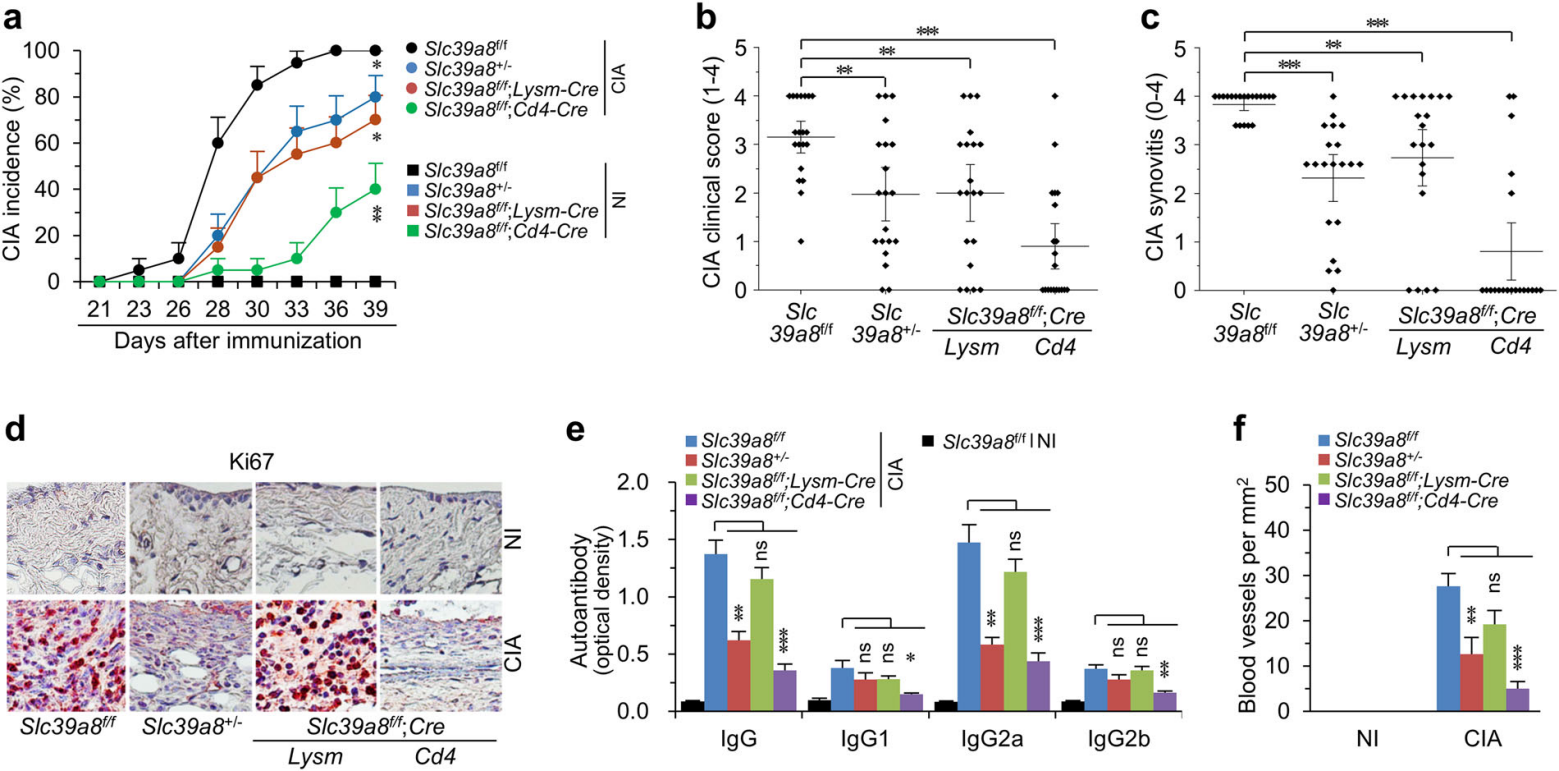
ZIP8 depletion in CD4^+^ T cells blocks CIA in mice. **a** Cumulative CIA incidence on the indicated days after the first immunization of WT (*Slc39a8*^*f/f*^), *Slc39a8*^*+/−*^, *Slc39a8*^*f/f*^;*Lysm-Cre*, and *Slc39a8*^*f/f*^;*CD4-Cre* mice. **b** Clinical scores of *Slc39a8*^*f/f*^, *Slc39a8*^*+/−*^, *Slc39a8*^*f/f*^;*Lysm-Cre*, and *Slc39a8*^*f/f*^;*CD4-Cre* CIA mice. **c** Synovitis scoring of the indicated CIA mice. **d** Representative images of Ki67 staining to detect proliferating cells (*N* = 5 mice per group). **e** Type II collagen-specific autoantibody production under NI and CIA conditions in the sera of the indicated mice. **f** Numbers of blood vessels in the synovial tissues of the indicated mice under NI or CIA conditions. The numbers of mice were 20 (**a**, **b**, **c**) or 8 (**e**, **f**) mice per group. All CIA parameters in (**b**–**f**) were determined on day 39 after the first immunization. The values are presented as mean ± SEM, and the *χ*^2^ test was used to analyze the incidence of CIA on day 39 (**a**); the values are presented as mean ± 95% CI and were assessed with the Mann-Whitney *U* test (**b**, **c**); the values are presented as mean ± SEM and were assessed with ANOVA followed by Bonferroni’s post hoc comparison (**e**, **f**). ^*^P < 0.05, ^**^P < 0.005, ^***^P < 0.0005. ns: not significant.

To elucidate the function of ZIP8 in infiltrated CD4^+^ T cells and CD11b^+^ macrophages, we generated cell type-specific cKO mice: *Slc39a8*^f/f^;*CD4*-*Cre* and *Slc39a8*^f/f^;*Lysm*-*Cre* (Supplementary Fig. 4a). *Slc39a8*^f/f^;*CD4*-*Cre* mice exhibited striking reductions in CIA incidence, whereas less inhibition was observed in *Slc39a8*^f/f^;*Lysm*-*Cre* or *Slc39a8*^+/−^ mice than in *Slc39a8*^f/f^;*CD4*-*Cre* mice (Fig. 4a). Similarly, other CIA parameters were more markedly inhibited in *Slc39a8*^f/f^;*CD4*-*Cre* mice than in *Slc39a8*^f/f^;*Lysm*-*Cre* or *Slc39a8*^+/−^ mice. These parameters included CIA severity (Fig. 4b), paw swelling (Supplementary Fig. 4b, c), synovial inflammation and hyperplasia (Fig. 4c, d and Supplementary Fig. 4d), the production of autoantibodies against type II collagen (Fig. 4e), angiogenesis in the inflamed synovial tissues (Fig. 4f and Supplementary Fig. 4e), pannus formation (Supplementary Fig. 5a), RANKL expression, the formation of TRAP-positive multinucleated osteoclasts in the pannus (Supplementary Fig. 5b, c), and cartilage destruction (Supplementary Fig. 5d). These results collectively indicate that the upregulation of ZIP8 in CD4^+^ T cells is crucial for the development of CIA in mice. We additionally observed normal development of CD4^+^ T cells in *Slc39a8*^f/f^;*CD4*-*Cre* mice (Supplementary Fig. 6a–c), indicating that the inhibitory effects of T cell-specific ZIP8 depletion in CIA are not due to any alterations in T cell development.

### ZIP8 in CD4^+^ T cells regulates Th17 cell differentiation in CIA mice

**Fig. 5:**
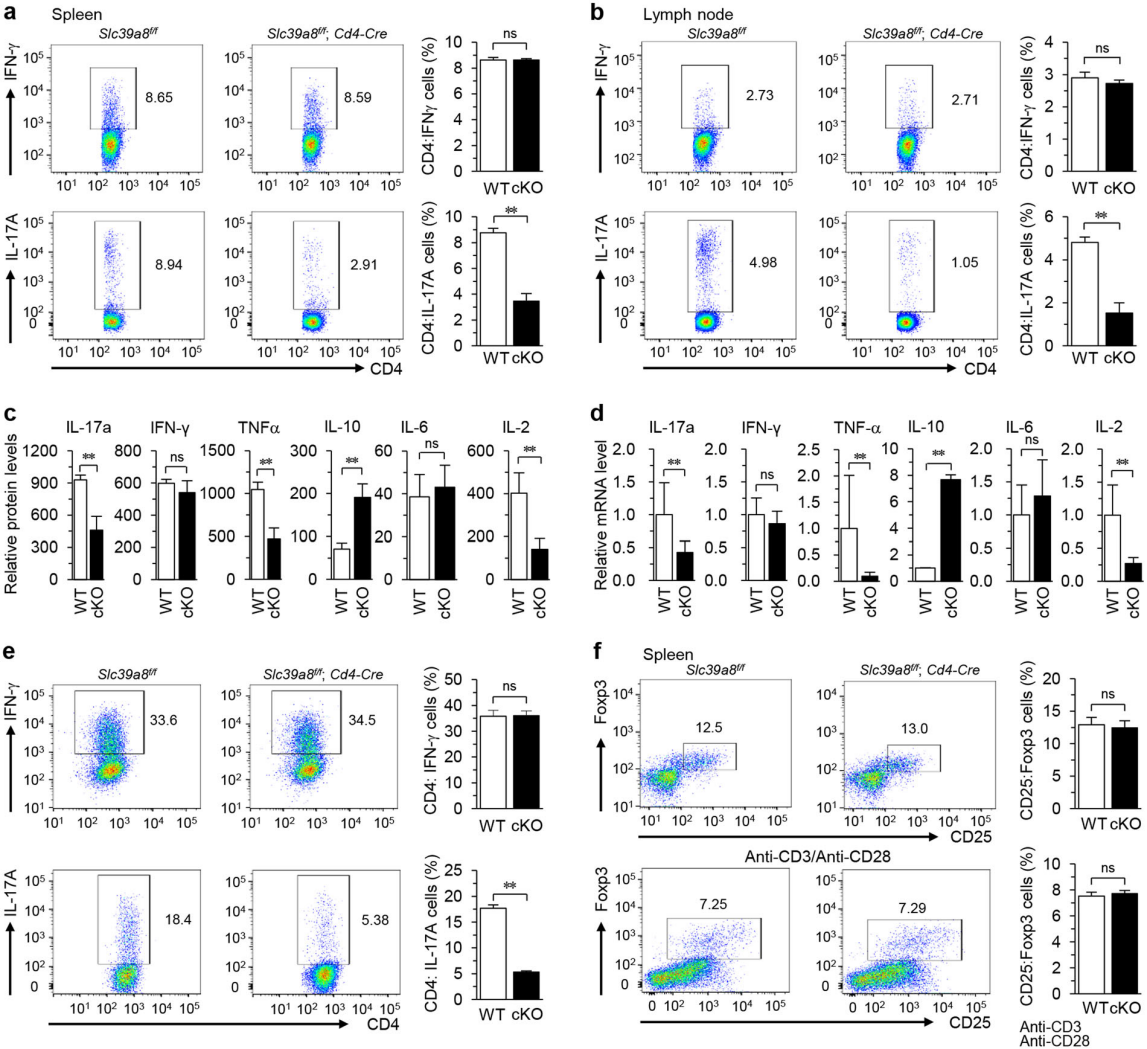
ZIP8 depletion in CD4^+^ T cells decreases the Th17 cell population in CIA mice. **a**, **b** Flow cytometric analysis of IFN-γ- or IL-17A-producing CD4^+^ T cells in the spleens (**a**) and lymph nodes (**b**) of *Slc39a8*^f/f^ and *Slc39a8*^f/f^;*CD4*-*Cre* mice on day 39 after the first immunization (*N* ≥ 4 mice per group). **c**, **d** Protein (**c**) and mRNA (**d**) levels of the indicated cytokines in the CD4^+^ T cells of *Slc39a8*^f/f^ and *Slc39a8*^f/f^;*CD4*-*Cre* mice on day 39 after the first immunization (*N* ≥ 6 mice per group). **e** Populations of Th1 and Th17 cells differentiated from uncommitted CD4^+^ T cells isolated from *Slc39a8*^f/f^ and *Slc39a8*^f/f^;*CD4*-*Cre* mice. **f** Tregs in the spleens of *Slc39a8*^f/f^ and *Slc39a8*^f/f^;*CD4*-*Cre* CIA mice (*N* ≥ 4 mice per group; upper panel). Treg population differentiated from naive CD4^+^ T cells isolated from *Slc39a8*^f/f^ and *Slc39a8*^f/f^;*CD4*-*Cre* mice (bottom panel). The data shown in (**e**) and (**f**) are representative of three independent experiments. The values are presented as mean ± SEM and were assessed with unpaired two-tailed Student’s *t*-tests. ^*^P < 0.05, ^**^P < 0.005. ns: not significant.

Compared with WT (*Slc39a8*^f/f^) mice, ZIP8-deficient *Slc39a8*^f/f^;*CD4*-*Cre* mice exhibited significant decreases in the population of IL-17A-producing but not IFN-γ-producing CD4^+^ T cells in both the spleen (Fig. 5a) and lymph nodes (Fig. 5b) under CIA conditions. In addition, the numbers of CD4^+^ T cells were decreased in the spleen and lymph nodes of *Slc39a8*^f/f^;*CD4*-*Cre* mice compared to WT mice under CIA conditions (Supplementary Fig. 7). Cytokine production in CD4^+^ T cells from *Slc39a8*^f/f^ and *Slc39a8*^f/f^;*CD4*-*Cre* mice under CIA conditions was analyzed and revealed that ZIP8 deficiency was associated with significant reductions in IL-17A, TNF-α, and IL-2 levels; increases in IL-10 production; and no significant changes in IFN-γ and IL-6 (Fig. 5c, d). Moreover, the in vitro differentiation of Th17 but not Th1 cells was significantly inhibited by ZIP8 deficiency in *Slc39a8*^f/f^;*CD4*-*Cre* mice (Fig. 5e). However, ZIP8 deficiency in *Slc39a8*^f/f^;*CD4*-*Cre* mice did not affect the regulatory T cell (Treg) population in CIA mice or Treg differentiation in vitro (Fig. 5f). In addition, the differentiation of Th2 cells was not significantly affected by ZIP8 deficiency (Supplementary Fig. 8).

### ZIP8 is a key regulator of zinc influx in effector CD4^+^ T cells

**Fig. 6:**
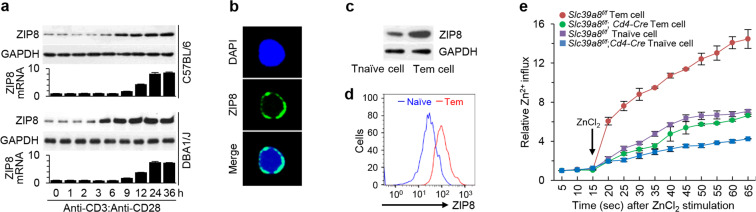
ZIP8 is a key regulator of zinc influx in effector CD4^+^ T cells. **a** CD4^+^ T cells were isolated from WT mice (C57BL/6 and DBA/1 J) and stimulated with 5 μg/mL anti-CD3 and anti-CD28 antibodies. The mRNA and protein levels of ZIP8 were determined at the indicated time points. **b** Representative confocal microscopic images of ZIP8 in primary cultures of mouse CD4^+^ T cells stimulated with anti-CD3 and anti-CD28 antibodies for 24 h. Nuclei were stained with 4,6-diamidino-2-phenylindole (DAPI). **c**, **d** Representative immunoblot image (**c**) and flow cytometric analysis (**d**) of ZIP8 in isolated CD4^+^ Tnaive and Tem cells. **e** Analysis of zinc influx in isolated Tnaive and Tem cells from *Slc39a8*^f/f^ and *Slc39a8*^f/f^;*CD4*-*Cre* mice. The arrow indicates the time of ZnCl_2_ treatment (45 μM). The data shown in (**a**–**e**) are representative of three independent experiments. The values are presented as mean ± SD. ^*^P < 0.05, ^**^P < 0.005.

The activation of CD4^+^ T cells from C57BL/6 or DBA1/1 J background mice was associated with marked increases in ZIP8 mRNA and protein levels (Fig. 6a). However, we did not detect any increase in ZIP6 during CD4^+^ T cell activation in our system (Supplementary Fig. 9a). The cell surface localization of ZIP8 on activated CD4^+^ T cells was confirmed by staining nonpermeabilized cells with an anti-ZIP8 antibody (Fig. 6b). We also observed increases in the total cellular level (Fig. 6c) and surface expression (Fig. 6d) of ZIP8 in Tem cells compared with Tnaive cells. Thus, ZIP8-mediated zinc influx may be an important regulatory mechanism for Tem cell activation. The role of ZIP8 in zinc influx was confirmed by measuring cellular zinc levels in Tem and Tnaive cells. Both cell types exhibited zinc influx after the addition of extracellular ZnCl_2_, and this influx was markedly inhibited by ZIP8 deficiency in *Slc39a8*^*f/f*^*;CD4-Cre* mice (Fig. 6e). Furthermore, consistent with their increased expression of ZIP8, Tem cells exhibited more notable zinc influx than Tnaive cells, and this influx was dramatically inhibited by ZIP8 deficiency (Fig. 6e).

### ZIP8 primarily regulates effector CD4^+^ T cell activation and proliferation through zinc flux

**Fig. 7:**
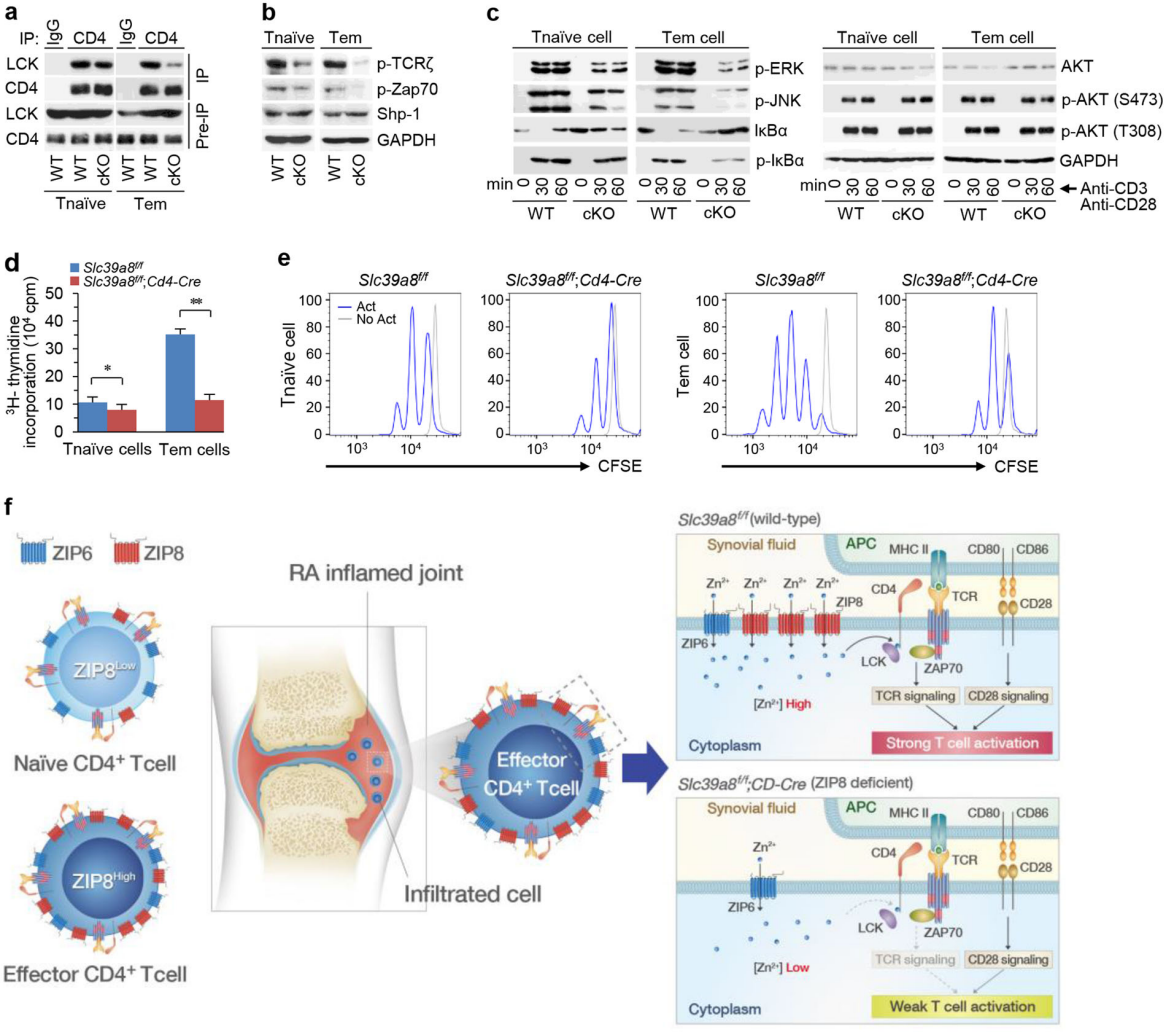
ZIP8 affects the effector stage of CD4^+^ T cell activation and proliferation. **a**–**c** Tnaive and Tem cells were isolated from WT (*Slc39a8*^f/f^) and cKO (*Slc39a8*^f/f^;*CD4*-*Cre*) mice and stimulated with or without anti-CD3 and anti-CD28 antibodies. Representative images of immunoblots used to detect the binding of CD4 to Lck (**a**), to detect phospho-TCRζ, phospho-Zap70 and Shp-1 (**b**), and to examine ERK and JNK phosphorylation and IκBα degradation (**c**). **d**, **e** The proliferation of Tnaive and Tem cells from *Slc39a8*^f/f^ and *Slc39a8*^f/f^;*CD4*-*Cre* mice stimulated with anti-CD3 and anti-CD28 antibodies was measured by [^3^H]thymidine incorporation (**d**) and CellTrace Violet (CTV) staining (**e**). The data in (**d**) were assessed with unpaired two-tailed Student’s *t*-tests. ^*^P < 0.05, ^**^P < 0.005. **f** The role of ZIP8 in CD4^+^ T cell-mediated RA pathogenesis. Effector CD4^+^ T cell activation and function require ZIP8 upregulation to increase zinc influx. Thus, ZIP8 depletion in CD4^+^ T cells blocked CD4^+^ T cell-mediated RA pathogenesis by inhibiting the supply of zinc to the enlarged CD4^+^ T cells.

Zinc is an essential component of TCR signaling during T cell activation^35^, and we examined the role of ZIP8 in TCR signaling. Whereas binding of Lck to CD4 was observed in ZIP8-expressing Tem and Tnaive cells, this binding was dramatically decreased in ZIP8-deficient Tem cells and slightly decreased in ZIP8-deficient Tnaive cells (Fig. 7a). ZIP8 deficiency was associated with decreases in the phosphorylation levels of TCRζ and TCR component zeta chain of TCR-associated protein kinase 70 (Zap70) in both cell types, and a more profound decrease was seen in Tem cells than in Tnaive cells (Fig. 7b). However, the expression level of Shp-1, which is a negative regulator of TCR signaling, was not changed. ZIP8 deficiency also modulated the phosphorylation levels of ERK, JNK, and IκBα and altered the degradation of IκBα in both Tem and Tnaive cells, with more marked effects observed in Tem cells than in Tnaive cells (Fig. 7c). Interestingly, ZIP8 deficiency did not affect AKT phosphorylation (Fig. 7c). As AKT is mainly involved in CD28-mediated signaling cascades^36^, our results indicate that ZIP8 may have specific functions in TCR signaling but not in the CD28-mediated signaling cascade. In addition to modulating TCR signaling, thymidine incorporation analysis revealed that ZIP8 deficiency significantly inhibited the proliferation of Tem and Tnaive cells, with more profound effects seen in Tem cells than in Tnaive cells (Fig. 7d). Similarly, cell division analysis by flow cytometry with CTV indicated that ZIP8 deficiency exerted more profound inhibitory effects on Tem cells than on Tnaive cells (Fig. 7e). We also found that depletion of extracellular zinc significantly inhibited the proliferation of activated CD4^+^ T cells (Supplementary Fig. 9b, c). Therefore, ZIP8-mediated zinc influx in Tem cells is important for activation and proliferation (Fig. 7f).

## Discussion

Specific types of zinc transporters, such as ZNT and ZIP family members, exhibit differential effects on various immune cells. Therefore, the regulation of zinc mobilization by cell type-specific ZNTs or ZIPs may be linked to the development of inflammatory diseases^19,20,22,23,37^. However, it has not yet been elucidated whether cell type-specific and transporter-specific zinc mobilization are involved in RA pathogenesis. Here, we investigated whether RA pathogenesis was associated with zinc mobilization by a specific zinc transporter in specific subtypes of immune cells. The present study showed that, as in RA patients^38^, zinc levels were locally increased in the synovial fluid and synovial tissues of CIA mice and that among all the examined zinc importers (ZIP1–ZIP14) and exporters (ZNT1–ZNT10), ZIP8 was specifically upregulated in the synovial tissues of inflamed joints in CIA mice and RA patients. We also demonstrated that ZIP8 was profoundly upregulated in infiltrated CD4^+^ T cells. Therefore, our current findings suggest that ZIP8-mediated zinc influx in infiltrated CD4^+^ T cells contributes to the onset and progression of CIA.

In this report, we also examined whether the zinc–ZIP8–MTF1–MT axis regulates CIA pathogenesis using *Slc39a8*^+/−^, *Mtf1*^+/−^, and *Mt1*^−/−^*Mt2*^−/−^ mice, as this axis is important in the pathogenesis of OA^26^. However, we found that *Slc39a8*^*+/−*^ mice exhibited strong inhibition of CIA, whereas reduced inhibitory effects were seen in *Mtf1*^*+/−*^ and *Mt1*^*−/−*^*Mt2*^*−/−*^ mice, and these findings were at odds with the pathogenic mechanism of OA. Therefore, our results indicate differences in the etiology and pathogenesis of RA and OA even though both conditions are regulated by ZIP8-mediated zinc influx. Different zinc-induced signaling pathways are, therefore, likely to be involved in OA and RA. Based on a previous report and current data, we suggest that ZIP8-mediated zinc influx into chondrocytes is important for OA pathogenesis by upregulating matrix-degrading catabolic enzymes^26,27^, while ZIP8 mediated RA pathogenesis primarily by modulating the functions of CD4^+^ T cells, such as Tem cell activation, including that of Th17 cells.

Our present results further demonstrate that ZIP8-mediated zinc influx regulates Tem cell activation and proliferation. Moreover, we found that depletion of extracellular zinc abrogates the in vitro proliferation and activation of CD4^+^ T cells. This observation is consistent with a previous report that an influx of extracellular zinc regulates CD4^+^ T cell activation^23^. ZIP8 appears to be an important zinc importer in CD4^+^ T cells that infiltrate inflamed joint tissues. Indeed, beyond our observation that ZIP8 was specifically upregulated in CD4^+^ T cells, we also found that the degree of zinc influx was proportional to the expression level of ZIP8 in these cells: ZIP8 expression was low in Tnaive cells, increased upon activation and was maintained at an elevated level in Tem cells. Consequently, more notable zinc influx was observed in Tem cells than in Tnaive cells, and the inhibitory effects of ZIP8 depletion on zinc influx were more profound in Tem cells than in Tnaive cells. Tem cells were larger than Tnaive cells. Thus, the increase in ZIP8 in Tem cells is essential for supplying zinc to maintain adequate zinc levels during the TCR signaling cascade. Therefore, we propose that ZIP8-mediated zinc influx in infiltrated CD4^+^ T cells is an important mechanism for regulating CD4^+^ T cell activation in the synovial region. We also found that ZIP8 deficiency significantly inhibited the differentiation of Th17 cells. Upon CD4^+^ T cell activation, ZIP8 expression levels were increased. Thus, during CD4^+^ T cell differentiation, ZIP8 depletion may inhibit Th17 cell differentiation by dysregulating TCR/CD28 signaling because TCR signaling was weakened while CD28 signaling was intact. A previous report showed that increasing CD28 signaling inhibited Th17 cell differentiation^39^. Thus, ZIP8 preferentially influenced Th17 cell differentiation, while the differentiation of other Th cells was not significantly affected by ZIP8. In addition, because Th17 cell differentiation is crucial for RA pathogenesis, including RANKL upregulation^1,2,5,40^, it was unsurprising that CIA development was abrogated in CD4^+^ T cell-specific cKO (*Slc39a8*^f/f^;*CD4*-*Cre*) mice. In addition, the total CD4^+^ T cell number was decreased in CIA-induced ZIP8-deficient mice, suggesting a role of ZIP8 in CD4^+^ T cell proliferation during CIA pathogenesis. Thus, our data suggest that ZIP8 expression in CD4^+^ T cells regulates the TCR signaling cascade and CD4^+^ T cell differentiation and proliferation during RA development and pathogenesis. However, it has also been reported that zinc supplementation inhibits Th17 cell differentiation^41^. Therefore, this finding suggests that the differentiation and function of Th17 cells can be inhibited at excessive or insufficient zinc concentrations. Thus, in addition to zinc supplementation, our data indicate that inhibiting certain zinc transporters may be another strategy for treating RA.

Zinc is an essential element for innate and adaptive immune responses^18^. Therefore, the elucidation of the roles of specific zinc transporters in immune cell functions could help us understand the molecular mechanisms underlying inflammatory diseases. Here, we provide evidence that inflammatory autoimmune diseases (i.e., RA) can be regulated by cell type- and transporter-specific zinc mobilization. Conclusively, ZIP8 is specifically upregulated in CD4^+^ T cells that infiltrate the inflamed synovial tissues of CIA joints and crucially contributes to CIA pathogenesis by regulating the activation and proliferation of effector CD4^+^ T cells, including Th17 cells. Our results collectively suggest that ZIP8 is a candidate therapeutic target for treating RA.

## Supplementary information

Supplementary materials.
