# Author Correction: ZIP8 exacerbates collagen-induced arthritis by increasing pathogenic T cell responses

**DOI:** 10.1038/s12276-025-01469-2

**Published:** 2025-07-15

**Authors:** Jung-Ah Kang, Ji-Sun Kwak, Sang-Heon Park, Kyu-Young Sim, Seul Ki Kim, Youngnim Shin, In Jung Jung, Jeong-In Yang, Jang-Soo Chun, Sung-Gyoo Park

**Affiliations:** 1 School of Life Sciences, Gwangju Institute of Science and Technology, Gwangju, 61005 Republic of Korea; 2 Cell Logistics Research Center, Gwangju Institute of Science and Technology, Gwangju, 61005 Republic of Korea; 3 Infectious Disease Research Center, Korea Research Institute of Bioscience & Biotechnology (KRIBB), Daejeon, 34141 Republic of Korea; 4 National Creative Research Initiatives Center for Osteoarthritis Pathogenesis, Gwangju Institute of Science and Technology, Gwangju, 61005 Republic of Korea; 5 College of Pharmacy, Seoul National University, Seoul, 08826 Republic of Korea

Correction to: *Experimental & Molecular Medicine*10.1038/s12276-021-00591-1, published online 01 April 2021

After online publication of this article, the authors noticed an error in the results section and supplementary information.

In the published paper, we discovered that an incorrect image was mistakenly included in Figure 4d (one of eight images) and in Supplementary Figure 4b. We sincerely apologize for this error.

Figure 4d

**Original** 
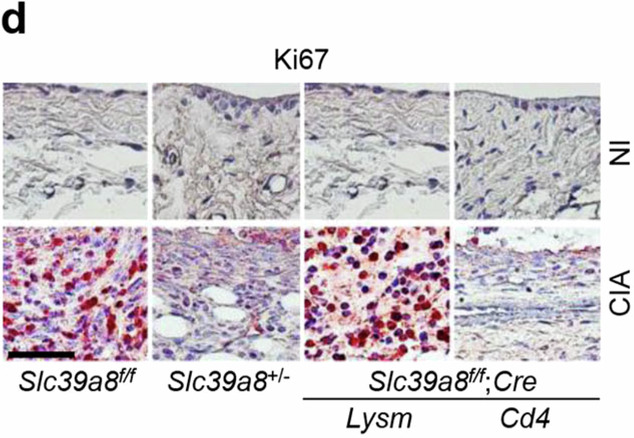


**Corrected** 
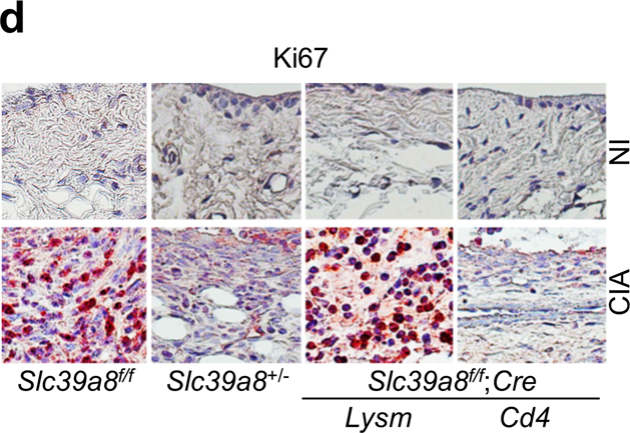


Supplementary Figure 4b

**Original** 
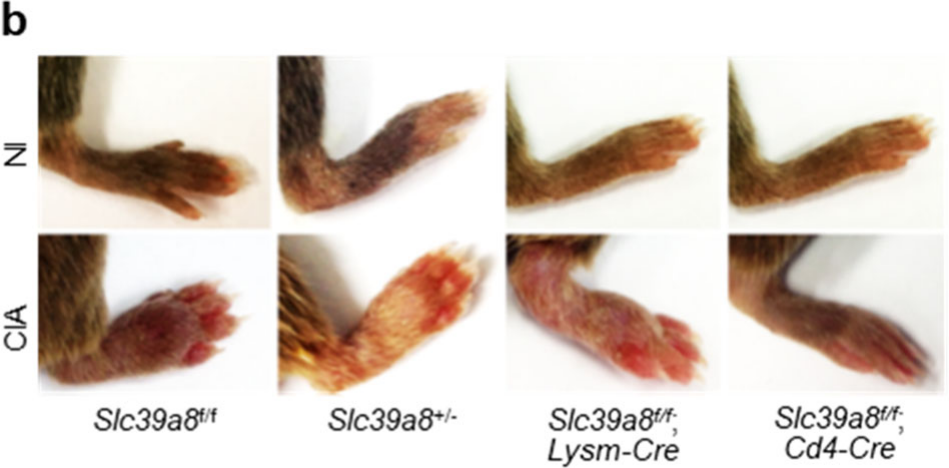


**Corrected** 
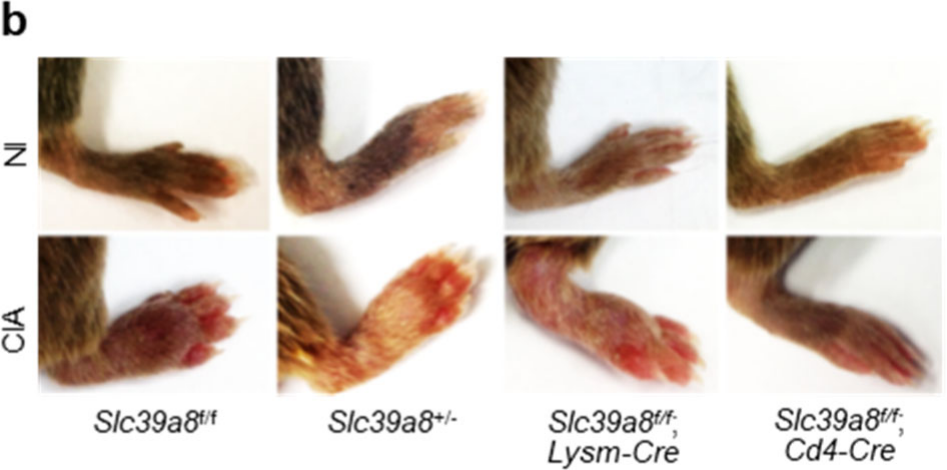


The authors apologize for any inconvenience caused.

The original article has been corrected.

## Supplementary information

Supplementary Figures

