# Supplementary material for: ZIP8 exacerbates collagen-induced arthritis by increasing pathogenic T cell responses
Source: Exp Mol Med. 2021 Apr 1;53(4):560–71. doi: 10.1038/s12276-021-00591-1 (PMC8102558; doi:10.1038/s12276-021-00591-1)
Supplement: Supplementary file 1 — Supplementary materials. [file 12276_2021_591_MOESM1_ESM.pdf]

Supplementary Figures

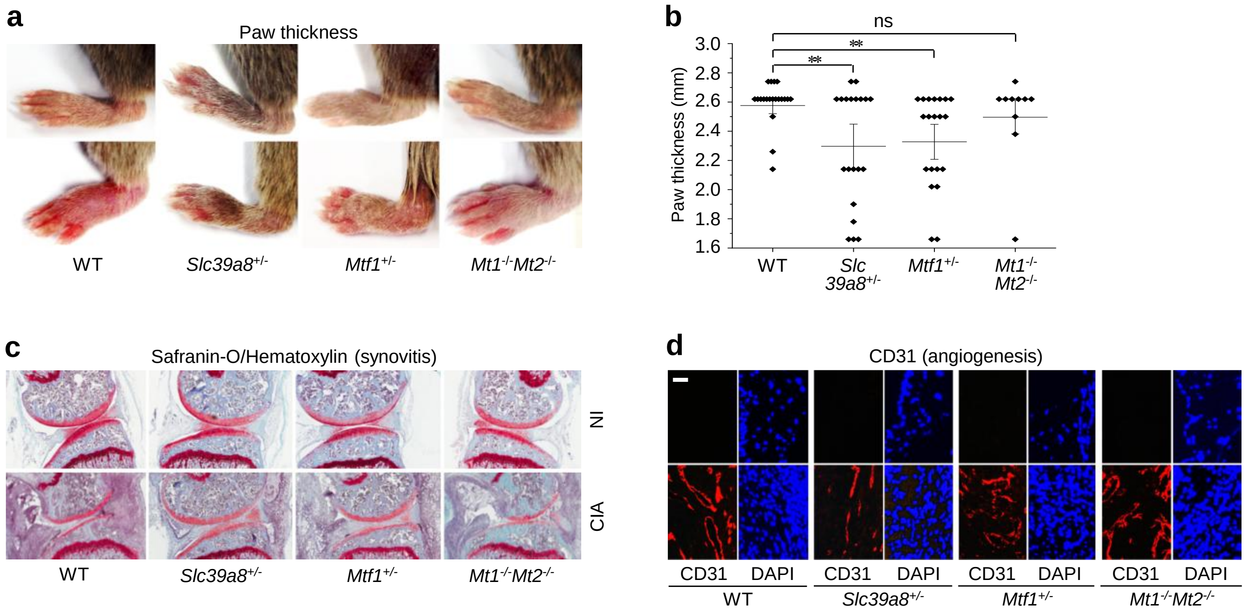

Supplementary Fig. 1 ZIP8 regulates synovitis and angiogenesis under the CIA condition. a, **b** Representative images of ankle paw (**a**) and paw thickness (**b**) of WT, *Slc39a8*$^{+/-}$, *Mtf1*$^{+/-}$, and *Mt1*$^{+/-}$*Mt2*$^{+/-}$ mice under the NI or CIA condition. c, d Representative images of safranin-O/hematoxylin staining of joint tissues to detect synovitis (**c**) and CD31 staining to detect blood vessels (**d**) in synovial tissues of WT, *Slc39a8*$^{+/-}$, *Mtf1*$^{+/-}$, and *Mt1*$^{+/-}$*Mt2*$^{+/-}$ mice under the NI or CIA condition. Numbers of mice used were 20 for WT, *Slc39a8*$^{+/-}$, and *Mtf1*$^{+/-}$ or 10 for *Mt1*$^{-/-}$*Mt2*$^{-/-}$. All CIA parameters were determined on day 36 after the first immunization. Values are presented as means ± SEM. and assessed with a two-tailed *t*-test. ** = $P < 0.005$, ns: not significant. Scale bar: 50 µm.

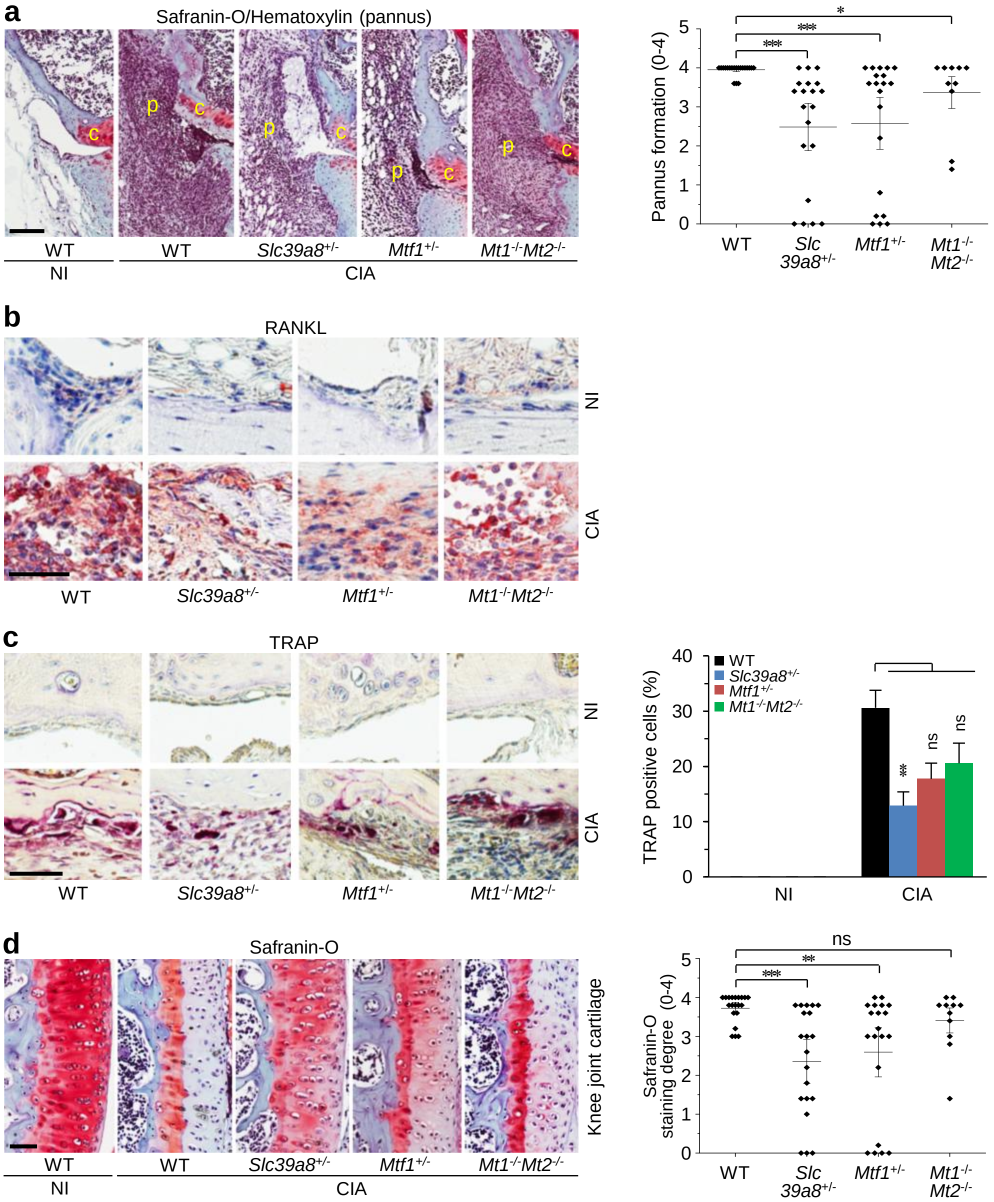

Supplementary Fig. 2 ZIP8 regulates pannus formation, bone erosion, and cartilage destruction under the CIA condition. **a** Representative images of pannus formation adjacent to cartilage and bone (left panel) and scoring of pannus formation (right panel) from WT, *Slc39a8*$^{+/-}$, *Mtf1*$^{+/-}$, and *Mt1*$^{+/-}$*Mt2*$^{+/-}$ mice under NI and CIA conditions. **b**, **c** Representative images of RANKL immunostaining (**b**), TRAP staining to detect multinucleated osteoclasts (**c**, left panel), and quantitation of TRAP-positive osteoclasts (**c**, right panel) in the pannus of the bone–cartilage interface under the NI or CIA condition. **d** Representative images of safranin-O staining to detect destruction of knee joint cartilage (left panel) and scoring of cartilage destruction (right panel) under NI and CIA conditions. Numbers of mice used were 20 for WT, *Slc39a8*$^{+/-}$, and *Mtf1*$^{+/-}$ or 10 for *Mt1*$^{-/-}$*Mt2*$^{-/-}$. All CIA parameters were determined on day 36 after the first immunization. Values are presented as means ± 95% CI and assessed with the Mann-Whitney *U* test (**a**, **d**) or means ± SEM. and assessed with ANOVA and Bonferroni's *post-hoc* comparison (**c**). * = $P < 0.05$, ** = $P < 0.005$, *** = $P < 0.0005$. ns: not significant. Scale bar: 50 μm.

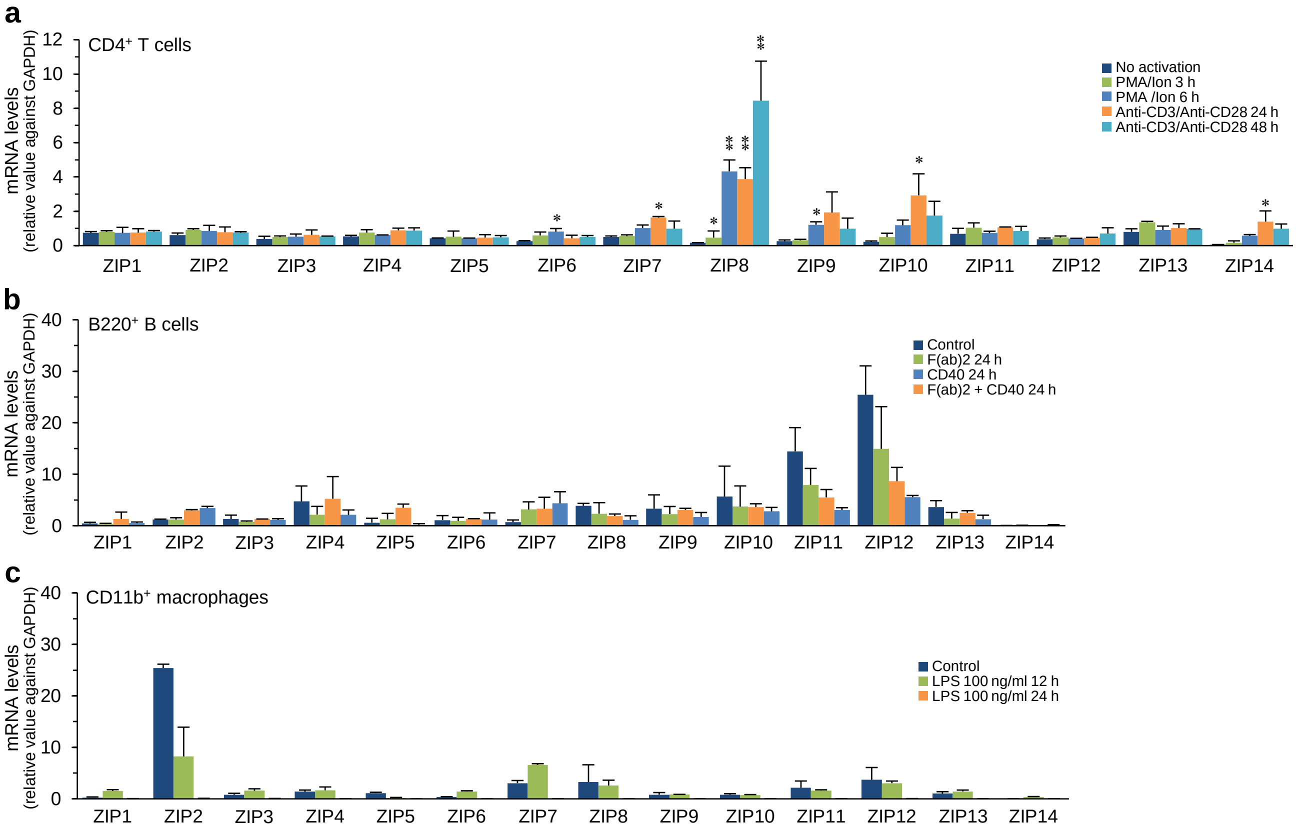

Supplementary Fig. 3 ZIP8 mRNA levels are increased in activated CD4$^+$ T cells. a–**c** Relative mRNA levels of ZIP family members were determined by qRT-PCR. CD4$^+$ T cells (**a**), B220$^+$ B cells (**b**), and CD11b$^+$ macrophages (**c**) were isolated from total synovial tissues with a FACSAria III cell sorter (BD Biosciences, >95% purity). CD4$^+$ cells were stimulated with PMA/ionomycin for 3 or 6 hours or with anti-CD3 (5 μg/ml) and anti-CD28 antibodies (5 μg/ml) for 24 or 48 hours. B220$^+$ cells were stimulated with anti-F(ab) (20 μg/ml) and/or anti-CD40 antibodies (10 μg/ml) for 24 hours. CD11b$^+$ cells were stimulated with 100 ng/ml of lipopolysaccharide (LPS) for 12 or 24 hours. The data shown in **a**–**c** are representative of three independent experiments. The error bars represent mean ± SD. and the data were assessed with the unpaired two-tailed Student's *t*-test. * = $P < 0.05$, **= $P < 0.005$.

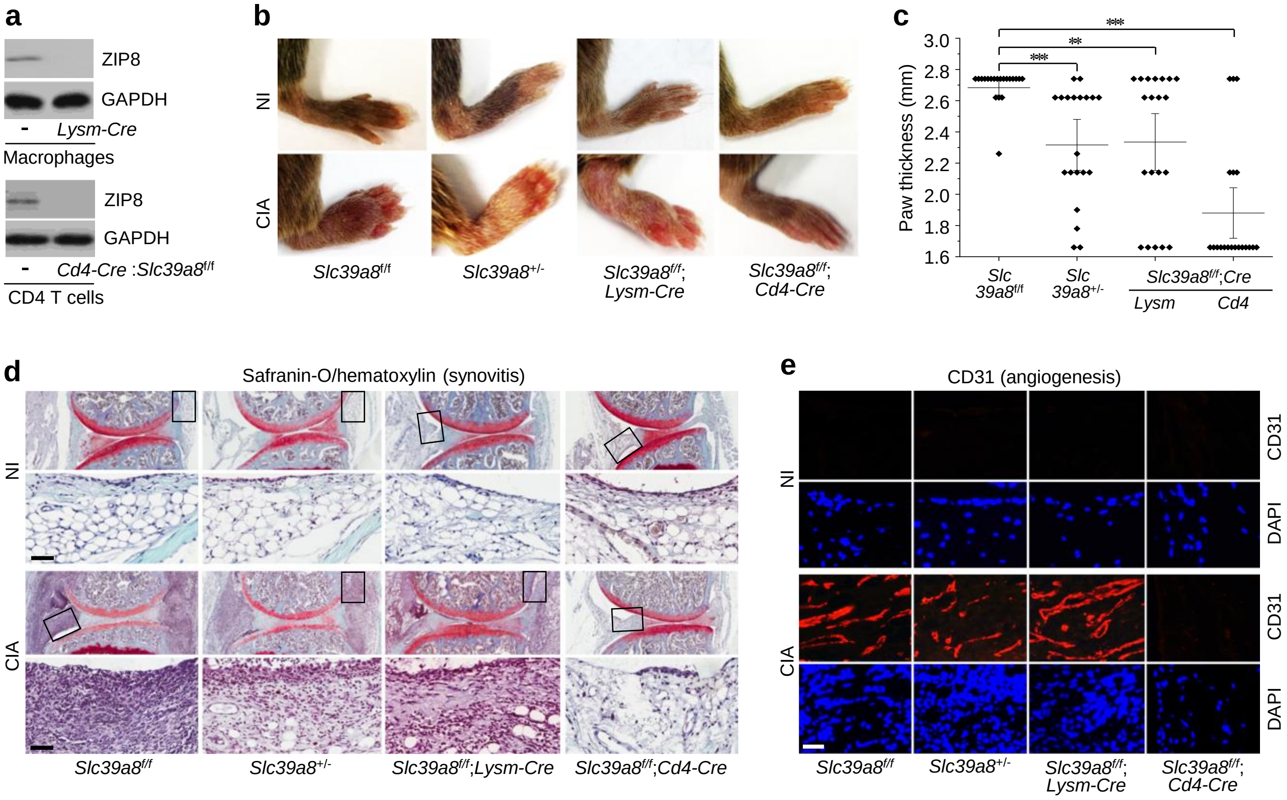

Supplementary Fig. 4 ZIP8 deficiency in T cells blocks synovitis and angiogenesis in mice under CIA condition. **a** Loss of ZIP8 expression was confirmed in macrophages isolated from *Slc39a8*$^{f/f}$;*Lysm-Cre* mice and CD4^{+} T cells from *Slc39a8*$^{f/f}$;*CD4-Cre* mice (n = 5). **b**, **c** Typical paw images (**b**) and paw thickness (**c**) of *Slc39a8*$^{f/f}$, *Slc39a8*$^{+/-}$, *Slc39a8*$^{f/f}$;*Lysm-Cre,* and *Slc39a8*$^{f/f}$;*CD4-Cre* mice under the NI or CIA condition (n = 20 mice per group). **d**, **e** Representative images (n = 20 mice per group) of safranin-O/hematoxylin staining to detect synovitis (**d**) and CD31 staining to detect blood vessels (**e**) in synovial tissues of *Slc39a8*$^{f/f}$, *Slc39a8*$^{+/-}$, *Slc39a8*$^{f/f}$;*Lysm-Cre,* and *Slc39a8*$^{f/f}$;*CD4-Cre* mice under the NI or CIA condition. All CIA parameters were determined on day 39 after the first immunization. Values are presented as means ± SEM. and assessed with the two-tailed *t*-test. ** =$P < 0.005$, *** = $P < 0.0005$. Scale bar: 50 μm.

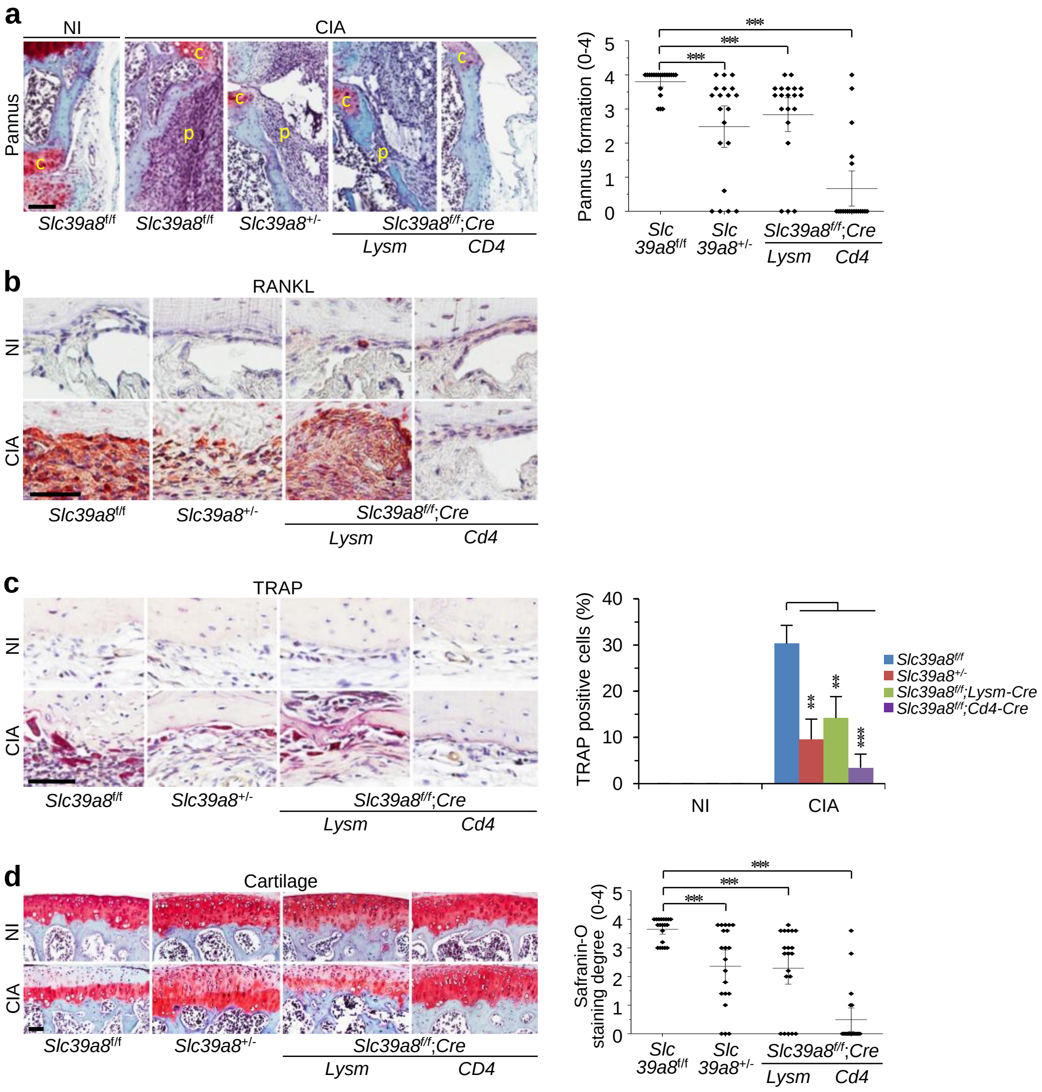

Supplementary Fig. 5 ZIP8 deficiency in T cells blocks bone erosion and cartilage destruction in mice under CIA condition. a Representative images (left panel) and scoring (right panel) of pannus formation adjacent to cartilage and bone (n = 20 mice per group) of *Slc39a8*$^{f/f}$, *Slc39a8*$^{+/-}$, *Slc39a8*$^{f/f}$;*Lysm-Cre*, and *Slc39a8*$^{f/f}$;*CD4-Cre* mice under the NI or CIA condition. **b**, **c** Representative images of RANKL immunostaining (**b**), TRAP staining to detect multinucleated osteoclasts (**c**, left panel), and quantitation of TRAP-positive osteoclasts (**c**, right panel) in the pannus of the bone–cartilage interface under the NI or CIA condition (n = 20 mice per group). **d** Representative images of safranin-O staining to detect cartilage destruction (left panel) and scoring of cartilage destruction (right panel) under NI and CIA conditions (n = 20 mice per group). All CIA parameters were determined on day 39 after the first immunization. Values are presented as means ± 95% CI and assessed with the Mann-Whitney *U* test (**a**, **d**) or means ± SEM. and assessed with ANOVA and Bonferroni's *post-hoc* comparison (***c***). ** = $P < 0.005$, *** = $P < 0.0005$. Scale bar: 50 μm.

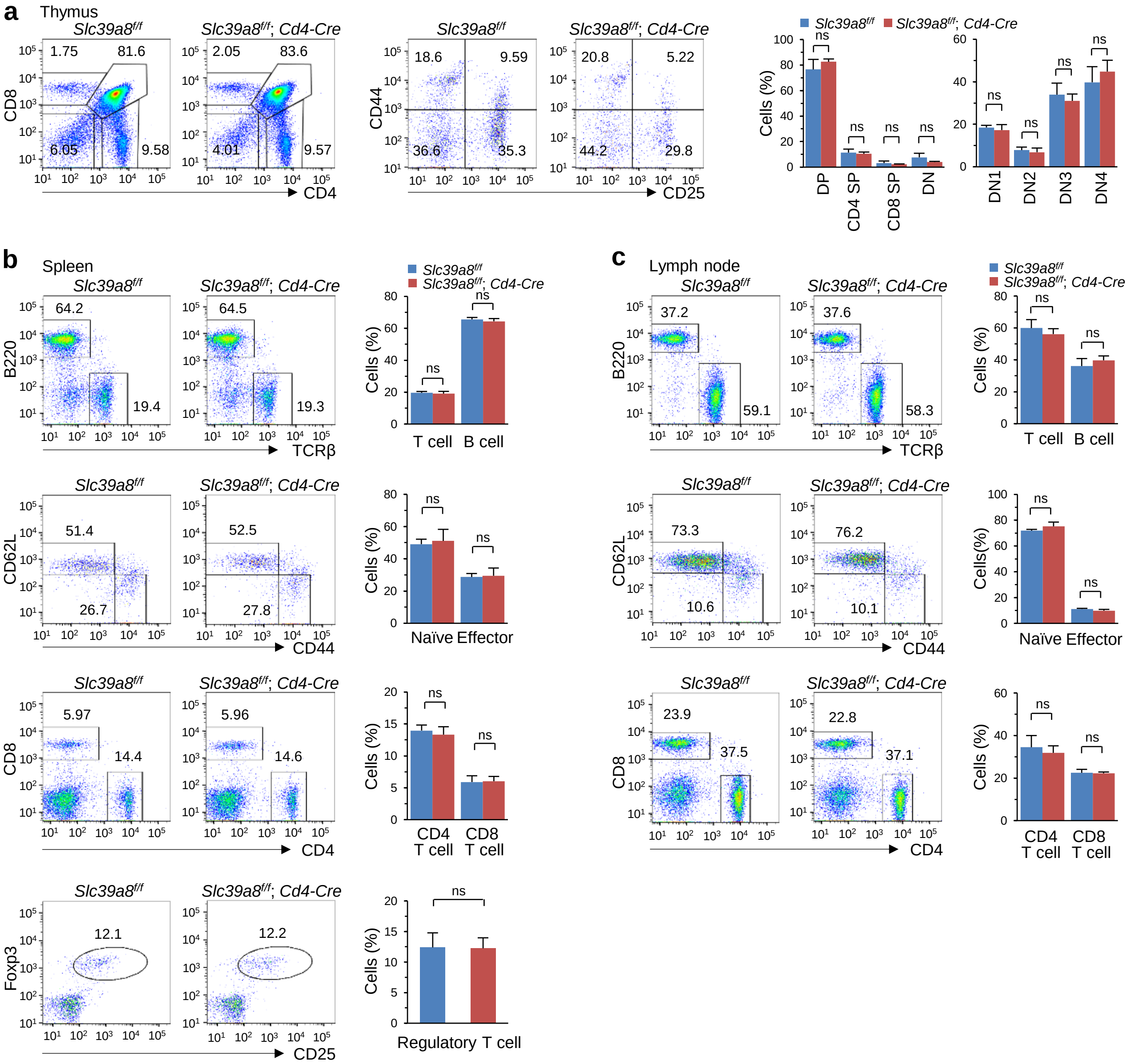

Supplementary Fig. 6 ZIP8 deficiency in developing T cells does not affect T cell development. a T cell development in the thymus was analyzed by flow cytometry. **b**, **c** Lymphocytes in the spleen (**b**) and lymph nodes (**c**) were analyzed by flow cytometry. Presented are representative results (n=3 mice per group); values are given as the mean ± SEM. and were assessed with the unpaired two-tailed *t*-test. ns, not significant.

DP, double-positive ($CD4^+CD8^+$); DN, doublenegative ($CD4^-CD8^-$); DN1, $CD44^+CD25^-CD4^-CD8^-$; DN2, $CD44^+CD25^+CD4^-CD8^-$; DN3, $CD44^-CD25^+CD4^-CD8^-$; DN4, $CD44^-CD25^-CD4^-CD8^-$.

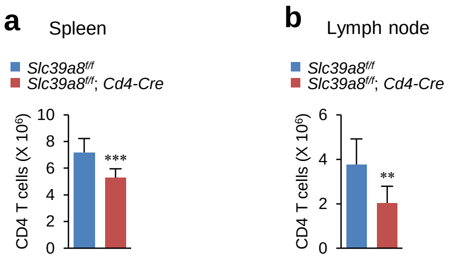

Supplementary Fig. 7 ZIP8 deficiency decreases CD4^{+} T cell in mice under CIA condition. Total cells were isolated from spleen (**a**) and lymph nodes (**b**) and CD4^{+} T cell number was counted by flow cytometry analysis (n≥5 mice per group). Values are presented as the mean ± SEM. and were assessed with the unpaired two-tailed *t*-test. ** = $P < 0.005$, *** = $P < 0.0005$.

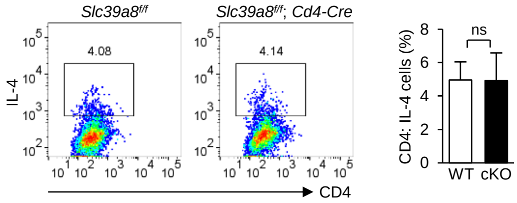

Supplementary Fig. 8 Populations of Th2 cells differentiated from uncommitted CD4$^+$ T cells isolated from *Slc39a8*$^{f/f}$ and *Slc39a8*$^{f/f}$;*CD4-Cre* mice. The data shown in this figure are representative of three independent experiments. Values are presented as the mean ± SEM. and assessed with the unpaired two-tailed Student's *t*-test. ns, not significant.

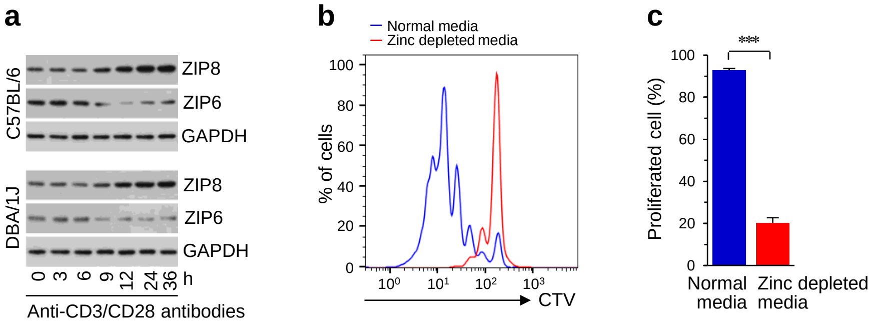

Supplementary Fig. 9 ZIP8, but not ZIP6, is upregulated during T cell activation. a $CD4^+$ T cells were isolated from WT mice (C57BL/6 or DBA/1J) and stimulated with anti-CD3 (5 μg/ml) and anti-CD28 (5 μg/ml) antibodies for the indicated time period. Presented are typical immunoblot images of ZIP8 and ZIP6. **b**, **c** The isolated $CD4^+$ cells were stained with CTV (CellTrace™ violet), stimulated with anti-mouse CD3 (5 μg/ml) and anti-mouse CD28 (5 μg/ml) antibodies, and incubated in normal or zinc-depleted medium for 72 hours. Values are presented as the mean ± SEM. and were assessed with the unpaired two-tailed *t*-test. *** = $P < 0.0005$.
